# Supplementary material for: Laterally gated ferroelectric field effect transistor (LG-FeFET) using α-In2Se3 for stacked in-memory computing array
Source: Nat Commun. 2023 Oct 25;14:6778. doi: 10.1038/s41467-023-41991-3 (PMC10600126; doi:10.1038/s41467-023-41991-3)
Supplement: Supplementary file 1 — Supplementary Infomation [file 41467_2023_41991_MOESM1_ESM.pdf]

Supplementary Information

Laterally Gated Ferroelectric Field Effect Transistor (LG-FeFET) using α-In_2Se_3 for Stacked In-Memory Computing Array

Sangyong Park[1,2†], Dongyoung Lee [3†], Juncheol Kang[3], Hojin Choi[3], and Jin-Hong Park[3]*

[1] *Flash Technology Development Team, R&D Center, Device Solutions, Samsung Electronics Co. Ltd, Hwasung, 18448 Korea*
[2] *Department of Semiconductor and Display Engineering, Sungkyunkwan University (SKKU), Suwon 16419, Korea*
[3] *Department of Electrical and Computer Engineering, Sungkyunkwan University (SKKU), Suwon 16419, Korea*
[†] *These authors contributed equally to this work.*

[*] *Corresponding author's email address: jhpark9@skku.edu (J.-H. P).*

Supplementary Note1.

MAC operations are simply implemented by memory cells that are connected to common source lines (CSL) and the vector-matrix-multiply (VMM) is conducted by the parallel MAC operations in the artificial neural network (ANN). In the ANN, the proportional increment of the number of the input and output nodes results in the quadratic increment of the number of cells as shown in Fig. S1.

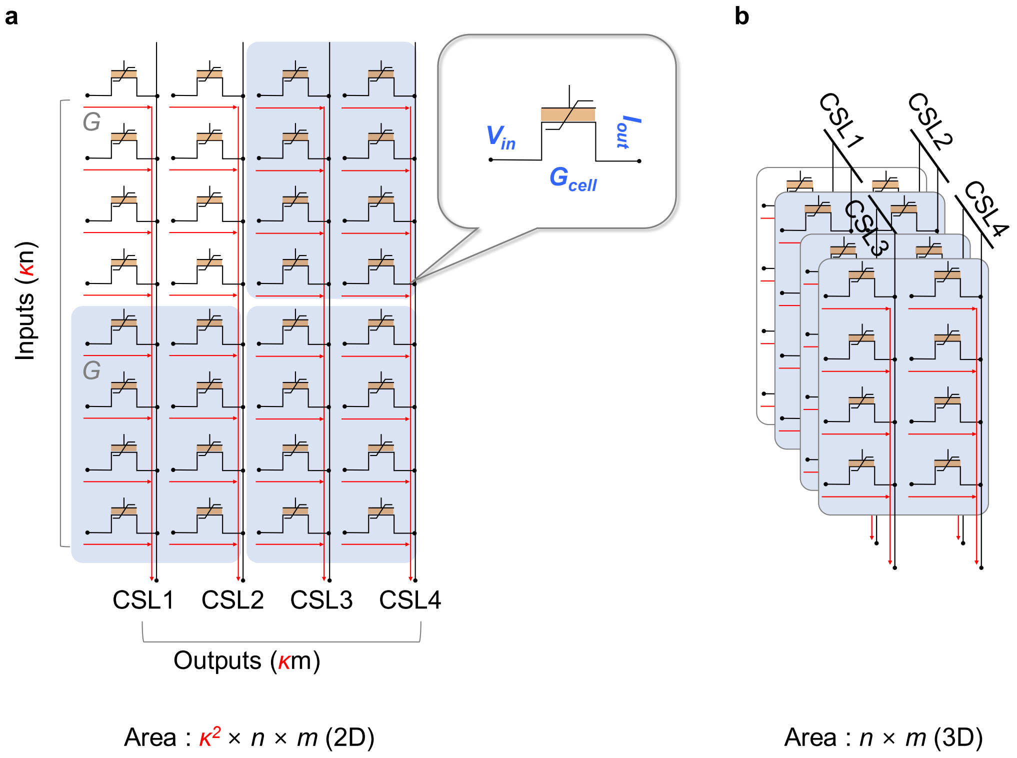

Supplementary Figure S1. Schematic images of two-dimensional and three-dimensional array structures. **a** The area increases by κ^2 times in a two-dimensional array structure as the number of rows and the columns increase by κ times. **b** In the stacked three-dimensional structure, cell density increases without increasing the area.

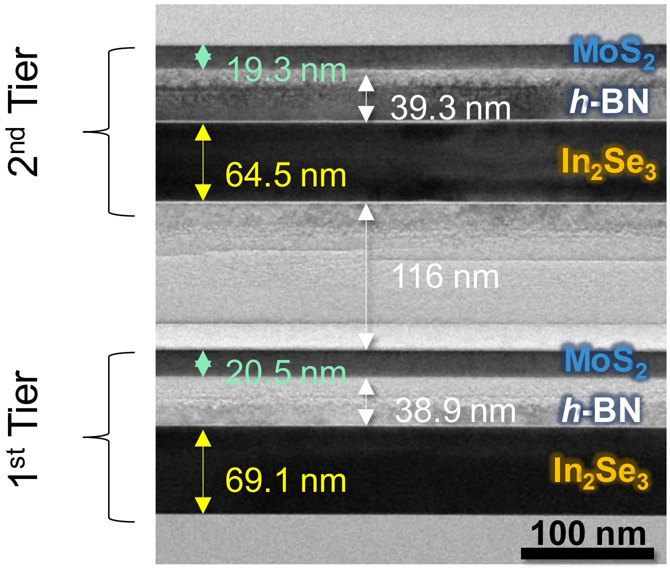

Supplementary Figure S2. Thicknesses of each layer including MoS_2, *h*-BN, and α-In_2Se_3.

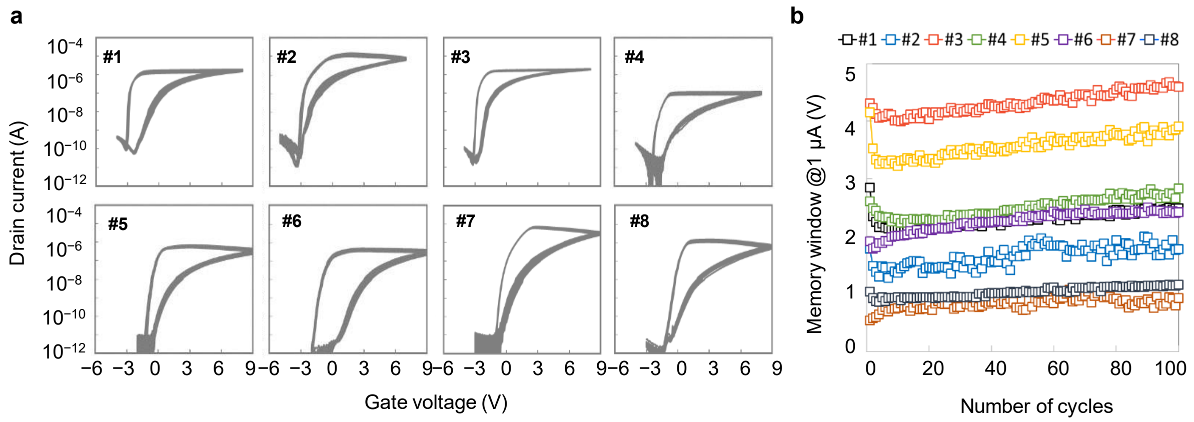

Supplementary Figure S3. a I_d-V_g transfer curves and **b** memory windows for eight different LG-FeFET devices throughout 100 double-sweep cycles.

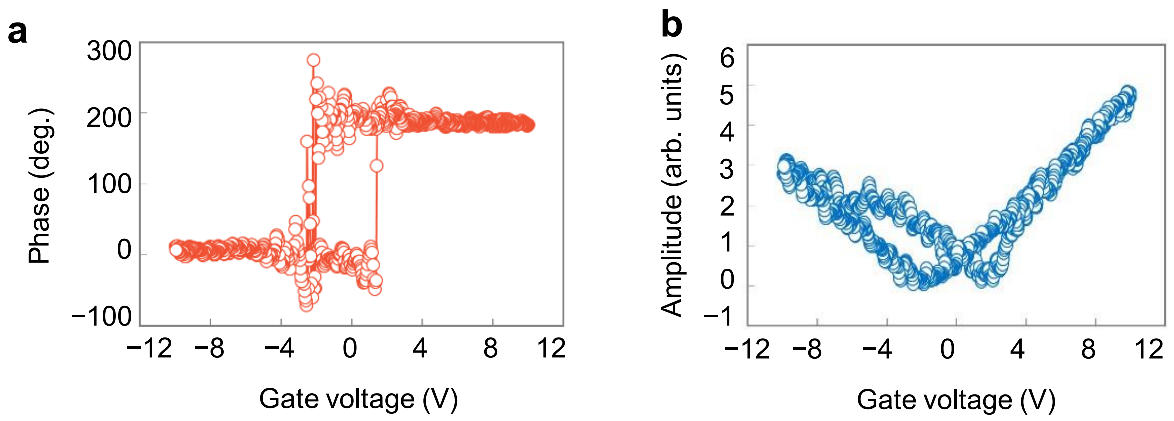

Supplementary Figure S4. Ferroelectric characteristics of α-In_2Se_3 analyzed by piezoelectric force microscopy (PFM). **a** The phase and **b** the amplitude show hysteresis and butterfly shapes according to the applied voltage.

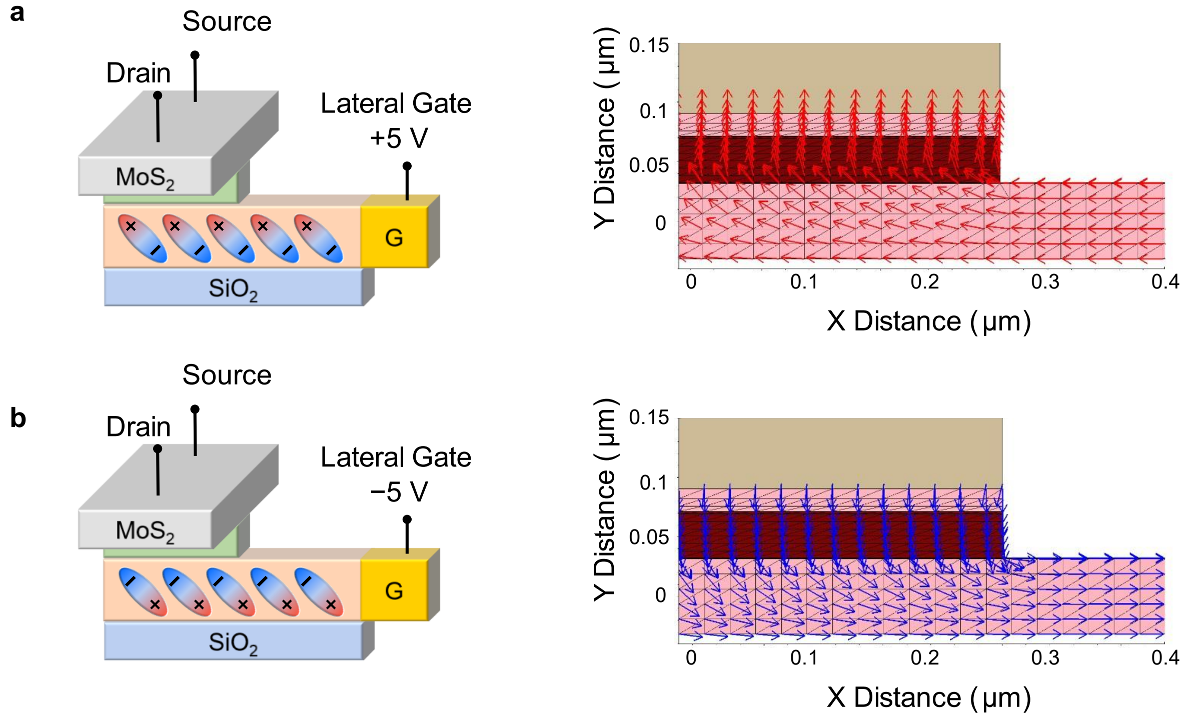

Supplementary Figure S5. Electric field vectors in the channel region when **a** a positive and **b** a negative gate voltage are applied to the lateral gate.

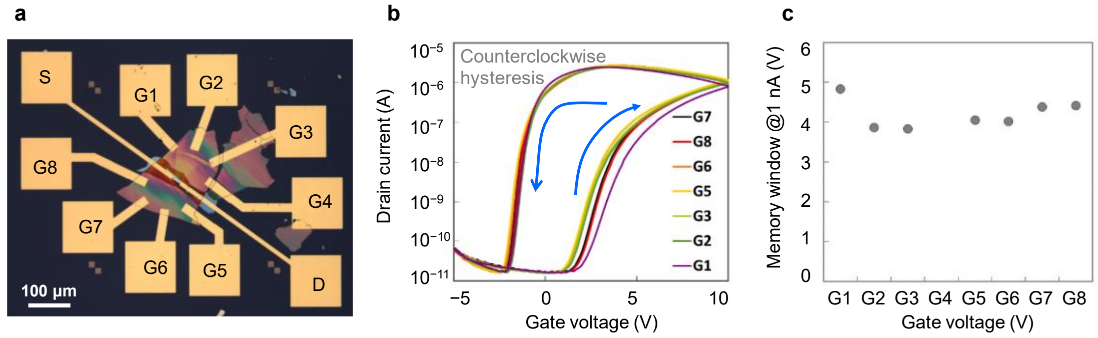

Supplementary Figure S6 a Optical microscopy image of the device which has eight gates around the channel region. **b** I_d-V_g transfer curves of the gates which is swept from −5 V to 10 V. All curves show counterclockwise hysteresis and hardly depends on the direction of the gates. **c** Memory windows for the various direction of the gates. (The memory windows are extracted at the 1 nA drain current.)

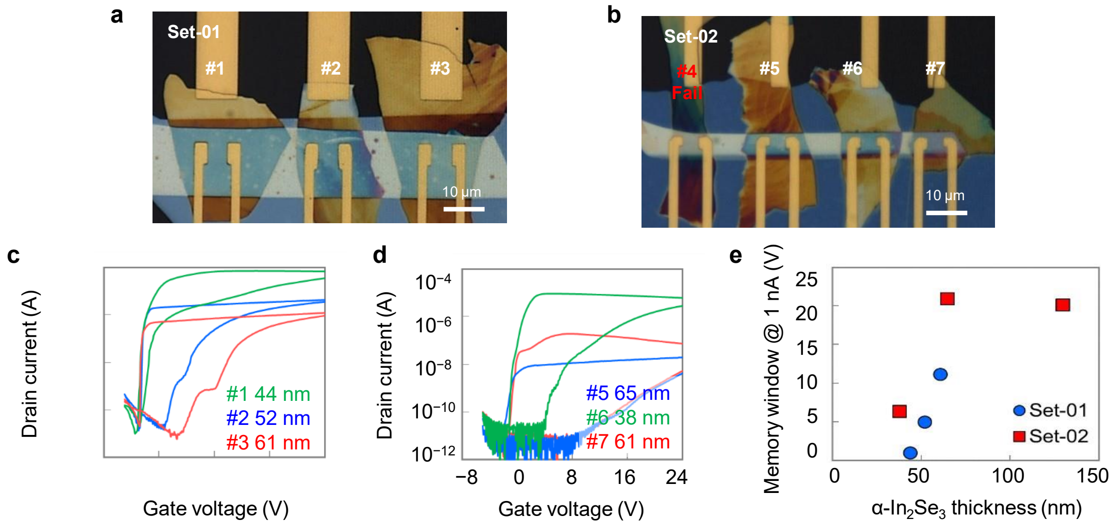

Supplementary Figure S7 a and **b** are the images of LG-FeFET devices. Each set shares the channel (MoS_2) and dielectric (*h*-BN) materials to minimize the variations caused by the TMD flakes. **c** and **d** represent the I_d-V_g transfer curves of set-01 and set-02, respectively. **e** illustrates the memory windows for various thicknesses.

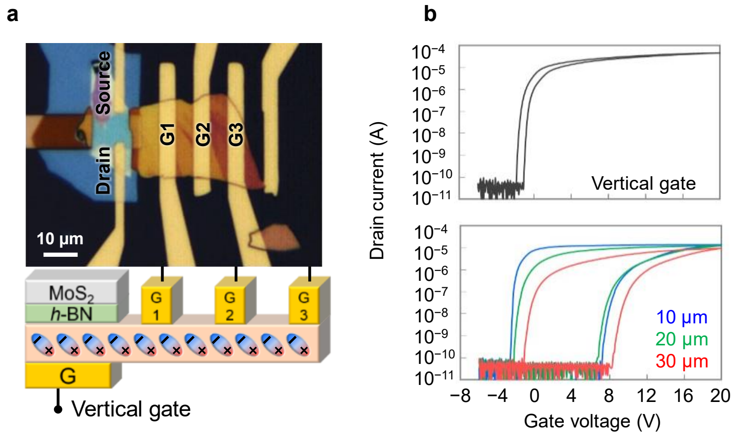

Supplementary Figure S8 **a** Optical microscopy image of the LG-FeFET device which has three different gate lengths. **b** I_d-V_g transfer curves of the vertical and lateral gates and **c** the memory windows. The lateral gates are positioned at distances of 10 μm, 20 μm, and 30 μm away from the channel area, respectively. All curves show counterclockwise hysteresis.

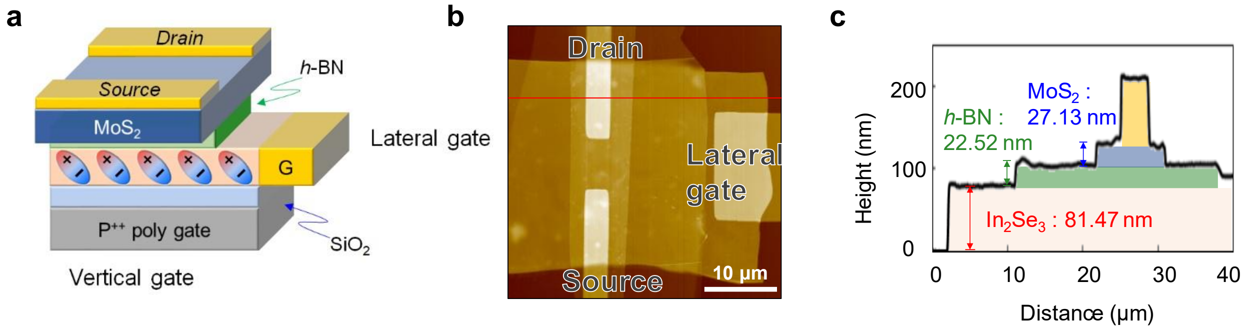

Supplementary Figure S9 **a** and **b** are the schematic and atomic force microscopy (AFM) image of the device, respectively. **c** is the vertical profile and thickness information which are obtained from AFM measurement.

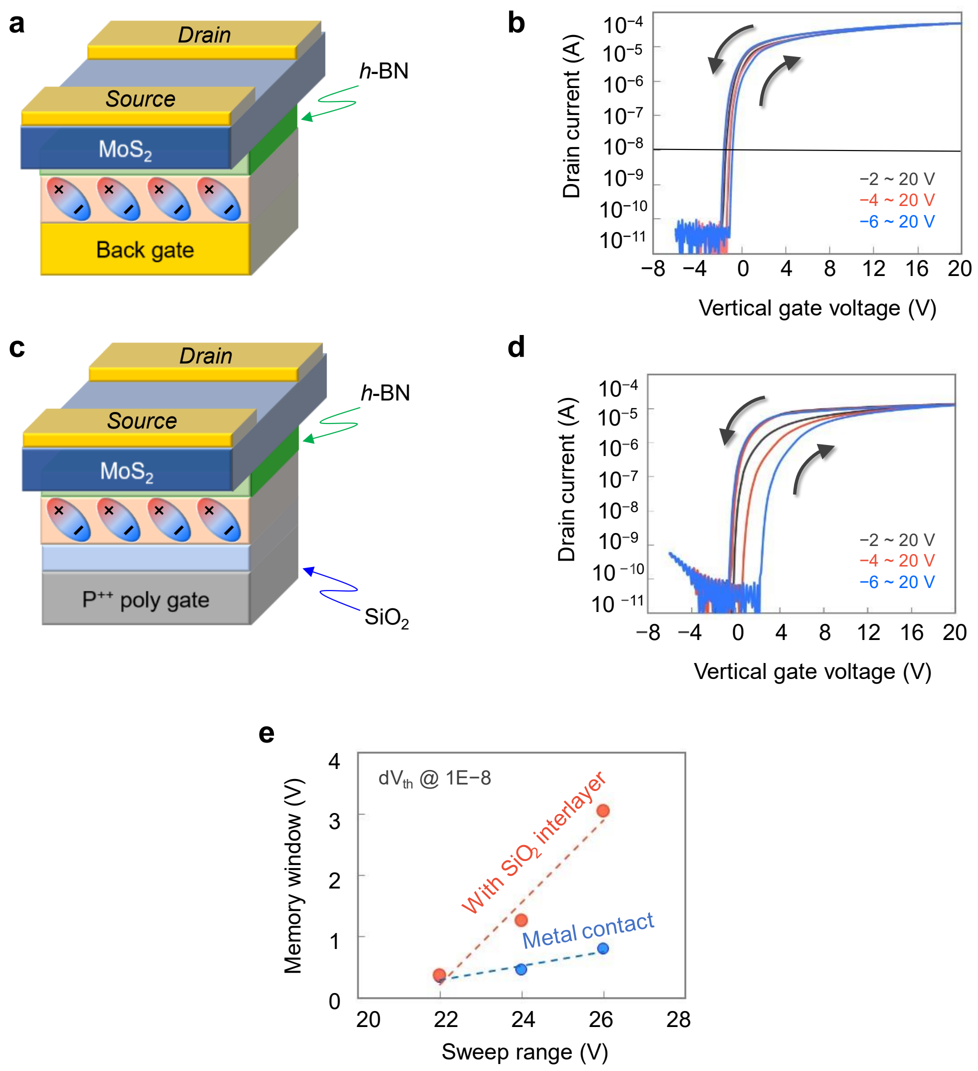

Supplementary Figure S10 Comparison of the effect of the interlayer between the vertical gate and ferroelectric layer. **a** and **b** show the device structure and I_d-V_g transfer curves for a device with directly contacted gate without an interlayer. **c** and **d** show the device structure and I_d-V_g transfer curves of the device with an interlayer between the vertical gate and the ferroelectric layer. **e** shows a comparison of the memory windows between the two devices.

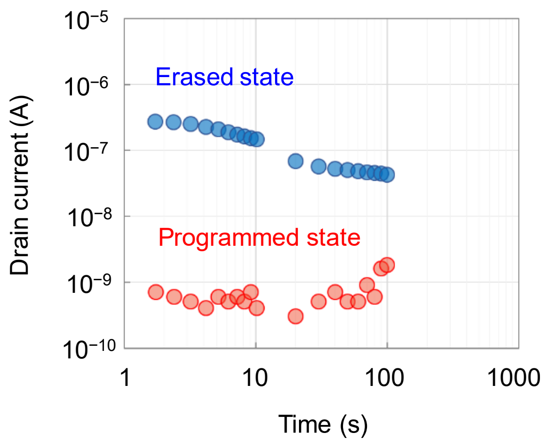

Supplementary Figure S11 Retention characteristics of an LG-FeFET device.

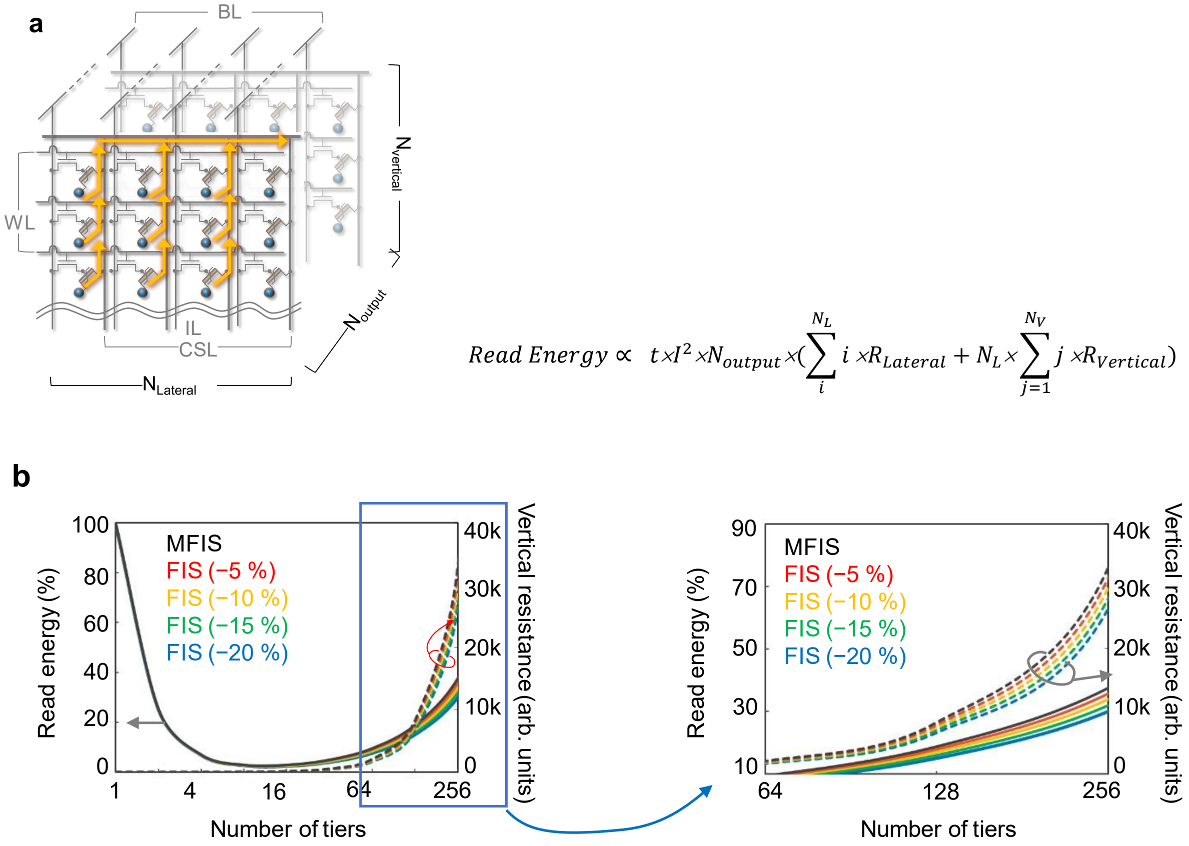

$$Read\ Energy \propto\ t \times I^2 \times N_{output} \times \left(\sum_{i}^{N_L} i \times R_{Lateral} + N_L \times \sum_{j=1}^{N_V} j \times R_{Vertical}\right)$$

Supplementary Figure S12 a show the equivalent circuit of the 3D stacked structure and the equation to estimate the energy consumption. **b** shows the reduction of read energy and the vertical resistance as a function of the number of tiers for the various portion of the metal gate. As the number of tiers increase, the read energy decreases due to the shorter lateral read path connected in series and the increased number of parallel connections. However, as the number of tiers continues to increase, the series resistance in the vertical direction starts to dominate the read energy, resulting in an increase of the read energy. The FIS structure of the LG-FET can mitigate the consumption of the read energy by reducing the vertical resistance.

Ref	Year	Author	Device Type	Interlocking effect	Polarization	Retention [sec]
[S1]	2020	Li.Y., et al.	2-terminal	O	IP	$>10^2$
[S2]	2019	Xue, F., et al.	2-terminal	O	IP	$>10^3$
[S3]	2021	Si. M,.	2-terminal	X	OOP	$>10^3$
[S4]	2019	S. Wan, et al.	3-terminal FeFET	X	OOP	$>10^4$
[S5]	2021	Dutta, D.	2-terminal	O	IP	$>3\times10^2$

Supplementary Table 1. List of the studies on the retention characteristic of α-In_2Se_3 ferroelectric memory.

Device	Hafnium-oxide based FeFET	LG-FeFET
Typical materials	X:HfO_2, HZO	α-In_2Se_3
Deposition	ALD, PVD	Exfoliation
Direction	Out-of-plane (OOP)	In-plane (IP)
Thickness	5 - 20 nm	40 - 130 nm
Memory window	1.5 - 3.5 V	About 10 V
Endurance	$<10^8$ cycles	$>10^5$ cycles
Retention	$>10^6$ s	About 10^4 s (Flake)<br>About 10^2 s (Device)
References	[S6]	This work

Supplementary Table 2. Comparison of the properties between an HZO-based FeFET and α-In_2Se_3 based LG-FeFET

	Sel./Unsel.				Word-Line (WL)	Bit-Line (BL)	Input -Line (IL)	Common-Source-line (CSL)
	**Plane**	**Cell**	**Sel.Tr.**	**Mem.Tr.**				
Inference operation	Sel.	Sel.	Sel.	Sel.	on	V_{read}	V_{input}	GND
		Drop-out	Unsel.	Unsel.	on	Floating	V_{input}	GND
	Unsel.	Un-sel.	Unsel.	Unsel.	off	V_{read}	V_{input}	GND
					off	Floating	V_{input}	GND
Learning operation	Sel.	Sel.	Sel.	Sel.	on	V_{update}	GND	GND
		Un-sel.	Unsel.	Unsel.	on	Floating	GND	GND
	Unsel.	Un-sel.	Unsel.	Unsel.	off	V_{update}	GND	GND
					off	Floating	GND	GND

Supplementary Table 3. Electrode conditions for inference and learning operations in 3D-stacked memory with LG-FeFETs.

Supplementary References

[S1] Li, Y. et al. Orthogonal electric control of the out-of-plane field-effect in 2D ferroelectric α-In_2Se_3. *Adv.Electron. Mater.* **6**, 2000061 (2020).

[S2] Xue, F. et al. Gate-tunable and multidirection-switchable memristive phenomena in a van der Waals ferroelectric. *Adv. Mater.* **31**, 1901300 (2019).

[S3] Si, M. et al. Asymmetric metal/α-In_2Se_3/Si crossbar ferroelectric semiconductor junction. *ACS Nano* **15**, 5689-5695 (2021).

[S4] Wan, S. et al. Nonvolatile ferroelectric memory effect in ultrathin α-In_2Se_3. *Adv. Funct. Mater.* **29**, 1808606 (2019).

[S5] Dutta, D., Mukherjee, S., Uzhansky, M. & Koren, E. Cross-field optoelectronic modulation via inter-coupled ferroelectricity in 2D In_2Se_3. *npj 2D Mater. Appl.* **5**, 44934 (2021).

[S6] Mulaosmanovic, H., et al. Ferroelectric field-effect transistors based on HfO_2: a review. *Nanotechnology* **32**, 502002 (2021).
